# A Review of Polymer Dielectrics for Redistribution Layers in Interposers and Package Substrates

**DOI:** 10.3390/polym15193895

**Published:** 2023-09-26

**Authors:** Pratik Nimbalkar, Pragna Bhaskar, Mohanalingam Kathaperumal, Madhavan Swaminathan, Rao R. Tummala

**Affiliations:** 1 3D Systems Packaging Research Center, Georgia Institute of Technology, Atlanta, GA 30332, USA; pragna@gatech.edu (P.B.); kmohan@ece.gatech.edu (M.K.); rtummala@ece.gatech.edu (R.R.T.); 2 Department of Electrical Engineering, Pennsylvania State University, University Park, PA 16802, USA; mvs7249@psu.edu

**Keywords:** advanced packaging, heterogeneous integration, polymer dielectrics, reliability

## Abstract

The ever-increasing demand for faster computing has led us to an era of heterogeneous integration, where interposers and package substrates have become essential components for further performance scaling. High-bandwidth connections are needed for faster communication between logic and memory dies. There are several limitations to current generation technologies, and dielectric buildup layers are a key part of addressing those issues. Although there are several polymer dielectrics available commercially, there are numerous challenges associated with incorporating them into interposers or package substrates. This article reviewed the properties of polymer dielectric materials currently available, their properties, and the challenges associated with their fabrication, electrical performance, mechanical reliability, and electrical reliability. The current state-of-the-art is discussed, and guidelines are provided for polymer dielectrics for the next-generation interposers.

## 1. Introduction

The amount of data generated and processed has seen an exponential increase in the past several years due to the digitization of systems. This trend is expected to accelerate further with the advent of generative Artificial Intelligence (AI). To handle such large amounts of data, system bandwidth needs continuous improvements. The CPU-memory bandwidth is expected to double every two years [1]. Interconnections between various chips are the main bottleneck that needs innovative solutions. There is a need to develop low-power, high-speed on-package copper wiring to tackle this challenge. Several new packaging architectures have been developed to cater to this need. Two main approaches include-(1) planar 2D structures such as interposers, where chips are placed next to each other, and (2) 3D architectures, where chips are stacked on top of each other. These approaches utilize either silicon dioxide or polymers as dielectric layers between the wiring for interconnections. Polymer-based wiring in organic interposers is fabricated using panel-manufacturing tools and processes and can support high input–output (IO) densities, as demonstrated by Shinko and Kyocera [2,3]. Forming these high-IO-density interconnects is highly dependent on the dielectric material properties and processing techniques. The pitch scaling of organic laminates is limited because of the dimensional stability of the core material. Non-polymer-based wiring uses silicon back-end-of-line (BEOL) infrastructure to achieve ultra-high IO densities as demonstrated by TSMC’s chip-on-wafer-on-substrate (CoWoS) [4] and Intel’s Embedded-Interconnect-Bridge (EMIB) [1]. However, non-polymer-based interconnects cannot support higher data rates because of the fundamental limitations of the dielectric.

**Figure 1:**
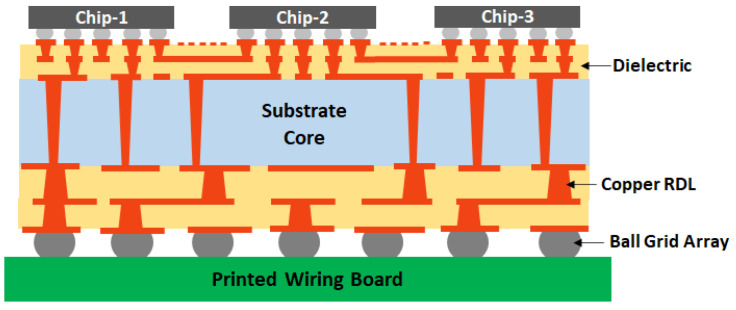
Schematic stack up of interposer/package substrate.

Figure 1 shows the schematic stack up of a package substrate or an interposer. A package substrate consists of a substrate core with multiple layers of polymer dielectric and copper wiring on either side. The copper wiring, also known as redistribution layers (RDLs), forms interconnections between the chips and the printed wiring board (PWB). There could be multiple packages stacked on each other as in the case of silicon interposers. Figure 1 depicts just one layer of package substrate for illustration. It should be noted that the terms “interposer” and “package substrate” are used interchangeably in this article due to the similar processing techniques utilized for the fabrication of both.

Table 1 lists the current state-of-the-art in 2.5D interposers and package substrate technologies. In general, wafer-scale technologies are able to achieve finer wiring because of the more-sophisticated damascene processing, but it utilizes silicon dioxide as the dielectric. A higher dielectric constant of silicon dioxide (D_*k*_ = 4) imposes limitations with respect to the maximum achievable data transfer rates. The effects of dielectric constant on electrical performance are discussed in detail in Section 3.

## 2. Fabrication and Processing

**Figure 2:**
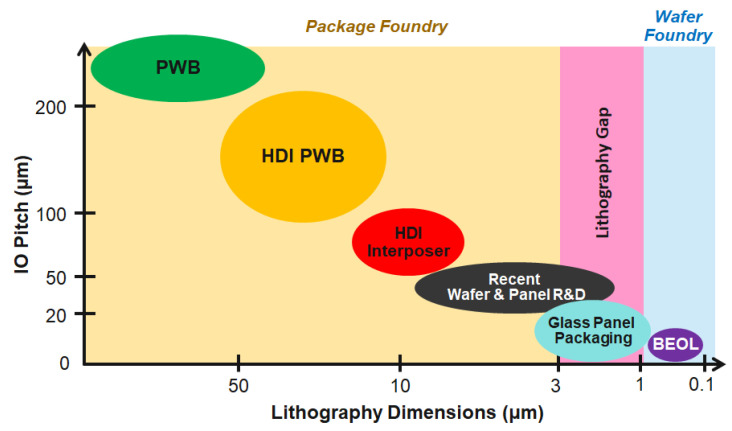
Package foundry bump pitch and lithography reaching Si wafer BEOL [16].

Figure 2 shows the trend in bump pitch and lithography dimensions for printed wiring board (PWB) substrates in comparison to BEOL wafer foundry. Panel-scale PWBs are produced at larger lithography dimensions (>50 μ m) in comparison to interposers. Panel-sized organic laminate substrates, until a few years ago, were produced at >10 μ m. There are several challenges faced by panel RDLs that limit the scaling of RDLs’ critical dimensions. Lithography tools for panels need a larger depth-of-focus compared to the BEOL counterparts, limiting the scaling to finer dimensions. Panel substrates also face dimensional abnormalities with respect to warpage and planarity and surface roughness, thus limiting the scaling. These surface topography deformations adversely affect the formation of fine features while patterning and worsen with multiple RDLs [12]. BEOL RDLs use dual-damascene processing to achieve finer RDL dimensions below 1 μ m up to 0.1 μ m. Because of the limitations of semi-additive processing and the lithography tools used in packaging, package RDLs are an order of magnitude larger than BEOL RDLs. This results in a lithography gap between the package and BEOL RDLs. However, many recent advances in wafer and panel packaging have extended package RDLs to 1 μ m. This has led to the bridging of the lithography gap depicted in Figure 2. BEOL RDLs, however, suffer from the disadvantage of higher cost due to smaller wafer-size processing. With recent advances in glass interposers, this issue has now been addressed by providing a panel-sized solution, thus lowering the cost of high-density substrates [13,14]. Additionally, panel-scale processing is critical for the manufacturing of large body-size interposers and substrates, which are less economical at the wafer scale.

Table 2 lists various polymer dielectrics commercially available on the market. The current state-of-the-art utilizes dry film (denoted as DF in Table 2) prepregs that are laminated onto substrates under the application of pressure and heat. Ajinomoto is the market leader with their several grades of Ajinomoto Buildup Films (ABFs), listed in Table 2. With the advances in interposer substrates, the trend is toward thinner dielectrics. This is one of the reasons for liquid dielectrics getting attention. Benzocyclobutene (BCB)-based dielectrics from Dow (trade name: Cyclotene) are popular and used mainly in wafer-scale packages due to the wafer-scale spin-coating of liquid dielectrics. Vapor-deposited dielectrics are also available on the market, but they are not popular for package substrates. Parylenes are the main class of materials that dominate this area. They are attractive for research for the next-generation interposers/substrates owing to their low dielectric constant values [15]. Several key properties of dielectrics are compiled and presented in Table 2 from the datasheets available online.

The selection of polymer dielectrics for packaging is challenging due to the conflicting nature of the fundamental physical properties of polymers. Usually, polymers having low dielectric constant (D_*k*_) values have high CTEs and low elastic moduli, as seen in Table 2. Traditional epoxy-based dielectrics used in package substrates have silica fillers that compensate for the poor mechanical properties of the polymer matrix, but result in an increased D_*k*_. Most low-k (D_*k*_ < 2.5) dielectrics do not have silica fillers, leading to lower D_*k*_, but poor mechanical stability. Thus, the selection of polymer dielectrics for RDL applications needs to be carried out with several considerations, which include thermal and mechanical stability, moisture sensitivity, processability, and chemical inertness. D_*k*_ and D_*k*_ need to be as low as possible to minimize RDL capacitance and dissipation losses. The processability of polymers is an important factor in being able to build a multilayer structure consisting of RDL routing with microvias. It is critical to control the thickness of the dielectric to achieve the desired microvia dimensions. For targeting smaller microvia dimensions, thinner dielectrics are needed. Dry film dielectrics conventionally used in package substrates are not available with less than a 5 μ m thickness. This leaves only liquid- or vapor-deposited dielectrics, as listed in Table 2. Another reason for desiring thin dielectrics is that a lower aspect ratio of the microvias gives better thermomechanical reliability. Liquid dielectrics are difficult to process on panels, as they need to be spin-coated. The uniformity and planarity of spin-coated dielectrics on large panels are poor, especially on multilayered substrates. Furthermore, for impedance matching, very fine thickness control (<1 μ m) is desired. Vapor-deposited polymers are, thus, very attractive due to sub-micrometer thickness control. Alternative processing techniques such as “slot die coating” are also gaining traction for depositing thin dielectric layers on large panels. The elastic modulus and CTE of polymers play crucial roles in the thermomechanical reliability of microvias. A low elastic modulus (<7 GPa) is desired to lower the stresses induced in RDLs. A very high modulus makes the polymer less pliable and not able to accommodate the expansion of copper. On the other hand, the CTE value needs to be as low as possible, preferably <40 ppm/K for RDL critical dimensions smaller than 5 μ m. Tensile strength and maximum elongation are also critical to prevent polymer cracking. Polymers become viscous and more flowable above their glass transition temperature. Therefore, to prevent RDL failure, the glass transition temperature should ideally be above the solder reflow temperature of about 250 °C. Moisture absorption in polymers leads to ionic migration under the influence of electrical bias. Therefore, moisture absorption needs to be <0.1% with ideally zero ionic content for conductor spacings of <2 μ m. In addition, the adhesion of the dielectric to copper is an important factor. This aspect will be discussed in detail in Section 4. Traditionally, package substrates are fabricated at lower temperatures than wafer RDLs. This is an important factor for keeping the cost of the substrates lower. Dielectrics with low curing temperatures <200° are, thus, desired.

**Figure 3:**
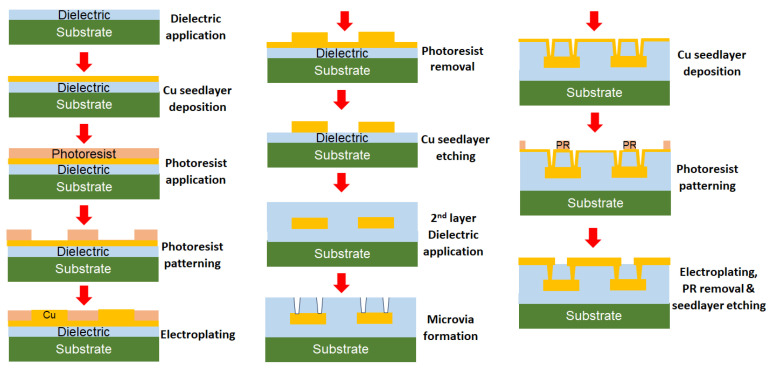
Multilayer semi-additive process flow for package substrate fabrication.

Figure 3 shows the various steps involved in substrate fabrication using a semi-additive process (SAP) traditionally utilized in panel-scale packaging. A core substrate is used to form multiple metal layers on either side. The polymer dielectric is applied using vacuum lamination of dry films followed by seed-layer metallization using electroless or sputter deposition. The next steps include photoresist patterning, electroplating, and photoresist removal. The seed layer used for metalizing is then etched away, thus completing the fabrication of one metal layer. For connecting the adjacent metal layers, vertical interconnects or microvias are drilled, conventionally using laser ablation. Alternatively, microvias can also be made using photo-imageable dielectrics (PIDs) using lithography. PIDs are gaining importance in the industry for their ability to form finer microvias, as well as lines with the damascene process. Figure 3 depicts only one-side processing; however, two-side processing is usually carried out for panel substrates. These steps are repeated to form multiple metal layers on the core to form an interposer or a package substrate. Traditional SAP involves wet processing techniques such as wet electroless deposition, photoresist development and stripping, and copper etching. The polymer dielectrics used to build up layers need to be resistant to the acidic and basic chemicals involved.

**Figure 4:**
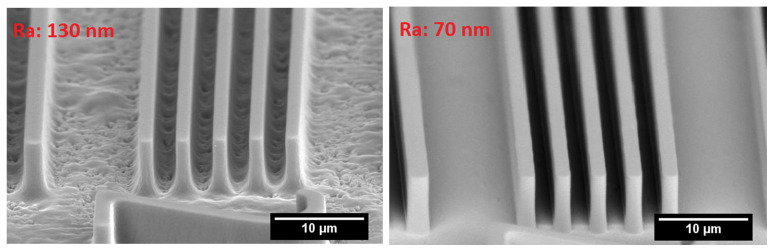
Effect of dielectric roughness on photoresist profile.

While there are many factors influencing adhesion, roughness is one of the main physical properties of the dielectric that influences the adhesion of copper lines onto the dielectric. However, higher roughness is detrimental to the resolution of fine-line features during lithography. Therefore, several materials having low surface roughness have been developed in order to achieve finer RDL wiring. Figure 4 demonstrates the effect that dielectric roughness can have in resolving fine line patterns in photoresists. Two dielectrics with average roughness values (Ra) of 130 nm and 70 nm were evaluated. A positive-tone photoresist was patterned with 2 μ m lines and spaces. A higher roughness results in diffused reflections at the copper–photoresist interface, resulting in residues and incomplete opening of the photoresist [17]. A smoother dielectric gives better results under the same conditions.

**Figure 5:**
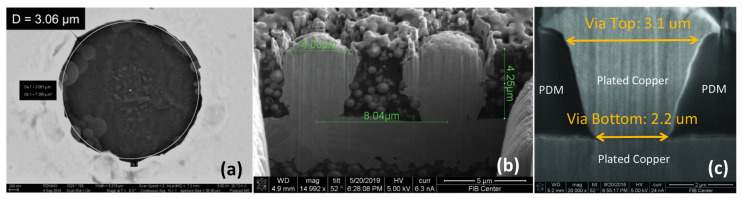
(**a**) Laser drilled microvia in ABF [19]; (**b**) tapered, Cu-filled microvia; (**c**) 3 μ m-diameter microvia formed in a photo-dielectric [20].

Miniaturization of microvias is important for maximizing the IO density of interposers. Figure 5 shows microvias formed in polymer dielectrics. Figure 5a shows a laser drilled microvia with 3 μ m diameter. Figure 5b shows a 4 μ m microvia filled with copper. The effect of the filler material on the via shape can be seen clearly. Smaller microvias are difficult to form in dielectrics with filler and often result in much debris, which creates processing challenges. Therefore, it is necessary to reduce the filler percentage, as well as the filler size for further microvia scaling. Figure 5c shows a microvia with a 3 μ m top diameter formed in a 5 μ m-thick PID using lithography. Tapering of vias is not desirable from the reliability perspective [18]. To achieve smaller and vertical microvias, thinner, filler-less, and higher-resolution PID materials are needed.

The traditional panel-scale SAP utilizes wet desmear followed by electroless deposition for seed layer deposition. Wet desmear involves roughening of the polymer surface using permanganate solution, which provides mechanical interlocking of the seed layer to the polymer. This, however, results in a very rough surface (Ra > 200 nm), leading to challenges during lithography for achieving <5 μ m critical dimensions. Therefore, there is an increased focus on the physical vapor deposition of metal seed layers for higher wiring density RDLs [21]. Layer-to-layer registration is a critical aspect affected by the CTE of the underlying dielectric. The CTE affects the dimensional shifts of the copper pads, thereby affecting the alignment accuracy of microvias. Low CTE and rigid dielectrics are, thus, desired for better via alignment accuracy [22].

## 3. Electrical Performance

Heterogeneous integration of chiplets is one of the main approaches adopted by the semiconductor industry to achieve higher performances at lower costs. In this approach, separately manufactured chiplets are integrated into an advanced interposer substrate. These heterogeneously integrated systems require high-bandwidth interconnections and low power consumption. Package parasitic losses need to be reduced for improving the signaling and electrical performance of the package. The low resistance and capacitance of RDLs are critical for reducing parasitic losses.

Bandwidth is driven by two factors—the number of IOs and the bit rate per IO (Equation (1)). The number of IOs is determined by the wiring density and the number of layers. The bit rate is determined by the signal speed and interconnect length. Therefore, in order to improve the bandwidth, faster data rates are needed.

(1) Bandwidth=IO/mm×Datarate/IO

**Figure 6:**
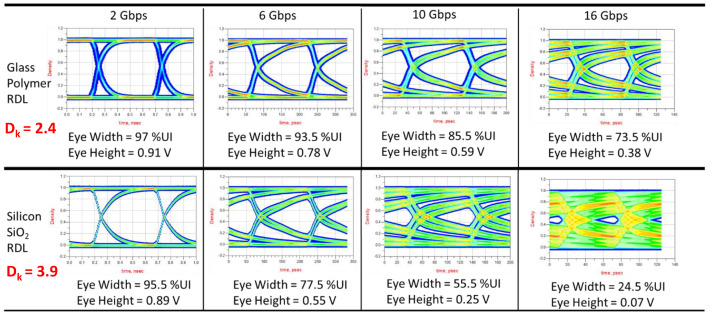
Effect of D_*k*_ on signaling performance [22].

Energy per bit (EPB) is an important metric used to compare the energy efficiency of heterogeneous systems. EPB is directly proportional to capacitance (Equation (2)), and achieving high energy efficiency requires the use of low D_*k*_ materials in interposers. There are studies indicating that a reduction in D_*k*_ from 3.9 to 2.4 can reduce the EPB by 40% for an interposer with an interconnect length of 5 mm [23]. The effect of D_*k*_ on the maximum achievable data rates can be seen in Figure 6. The readability of the signal degrades as the bit rate is increased from 2 Gbps to 16 Gbps. The eye width is just 24.5% of the unit interval at 16 Gbps for silicon RDLs due to the higher capacitance. Silicon-dioxide-based damascene RDLs, thus, have limitations in terms of increasing the data transfer rates.

(2) $$EPB=\frac{1}{2}C_{T}V_{swing}^{2}$$

Crosstalk, latency, and losses need to be reduced to improve the bandwidth density of RDLs. Crosstalk mainly originates from the capacitive coupling of metal wires, and losses arise from the resistance and capacitance of the wires. Latency is also a function of the resistance and capacitance of the channels. Therefore, reducing the capacitance of the dielectric leads to improvements in latency, bandwidth, and energy efficiency.

Traditional silicon back-end RDLs use silicon dioxide as a dielectric, which has a high dielectric constant (D_*k*_ = 4) and, thus, is limited in electrical performance. Therefore, polymer dielectrics with D_*k*_< 3 are gaining importance. The dielectric constant and loss are functions of frequency. In typical dielectrics, there are several types of polarization—orientation polarization, ionic polarization, distortion polarization, and electronic polarization [24]. All these are frequency-dependent, and their combined effect determines the dielectric constant at a certain frequency. The dielectric constant of polymers depends on the polarizability of the chemical bonds and groups present in them. Different chemical bonds have different polarizability. Organic molecules and bonds have low polarizability in general. Because of this, the lowest dielectric constant materials are generally polymers. Several polymers have been synthesized so far having low D_*k*_ for use in RDLs at the chip level [25]. However, wider adoption is absent due to the integration and reliability challenges.

**Figure 7:**
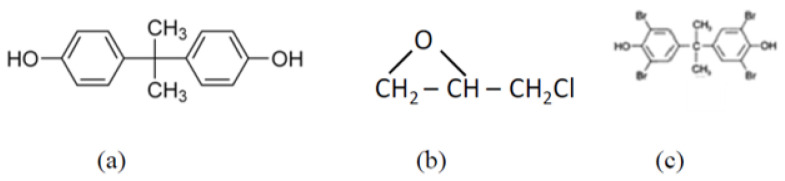
Structure of the main constituents of epoxy resins: (**a**) Bisphenol A (**b**) epichlorhydrin, and (**c**) bromine containing compound [27].

Table 3 shows the values of the polarizability and bond strength of common chemical bonds present in polymers [25]. The polarizability of sigma bonds is lower than that of pi bonds. Saturated hydrocarbon groups are, thus, desired in polymer dielectrics. However, higher bond strength is desired from the mechanical reliability point of view. Low polarizability also means low chemical reactivity of molecular groups, resulting in the poor processability of polymers. Because of these conflicting properties, a balance of various molecular groups is necessary to achieve the desired combination of electrical and mechanical properties of polymer dielectrics. The standard dielectric materials used in organic package substrates are based on epoxy polymers. Traditional epoxy polymers for packaging are primarily a blend of Bisphenol A and epichlorohydrin, shown in Figure 7a,b. The compound containing bromine shown in Figure 7c is added for fire retardancy, which is necessary for packaging applications. Different grades of Ajinomoto Buildup Film (ABF) are some examples of epoxy-based polymer dielectrics. Some epoxies also contain functional groups that are more polar, such as carbonyl and hydroxyl. While these functional groups are beneficial for improving adhesion to metals such as copper and are favorable from that standpoint, they often result in higher losses. Cyanate esters have low dielectric constants and low dielectric loss factors. These have been introduced in epoxy-based materials to reduce losses [26]. The presence of stiffeners in the main chain of the polymer such as phenyl rings limits the mobility of the structure and helps in achieving low loss [27].

## 4. Mechanical Reliability

A high-performance package substrate consists of multiple layers of polymer dielectrics and conducting metal lines. The reliability issues arise because of the inherent mismatch between the physical and mechanical properties of these layers. Polymer dielectrics have a higher CTE and a lower elastic modulus, whereas copper used for conducting lines has a relatively lower CTE and higher elastic modulus. This mismatch results in the development of stresses. These stresses are developed mainly during the fabrication processes and during the regular operation of electronic devices. Continuous buildup of the stresses ultimately results in physical deformations and, thus, permanent failure of the package and, thereby, the device. It is, therefore, important to design interposers to sustain the stresses developed during their targeted lifetimes. The thermal cycling test (TCT) and the highly accelerated stress test (HAST) are two main reliability tests performed on interposers.

### 4.1. Polymer–Cu Adhesion

The adhesion of copper to polymer dielectrics is the most-important issue pertaining to the miniaturization of RDL L/S. Delamination of copper traces from the dielectric becomes more probable at finer L/S because of the increase in stresses. Because of the presence of molecular groups having low polarizability, adhesion between the copper seed layer and low-D_*k*_ polymers is challenging. Low-D_*k*_ dielectrics have low roughness because of the absence of filler. Because of the smoother surface, adhesion by mechanical interlocking is insignificant. Therefore, it becomes essential to optimize or develop innovative pre-sputtering processes to enhance adhesion. There are several ways of enhancing adhesion reported in the literature [28]—surface roughening, chemical modification of the surface, use of an adhesion-promoting layer, UV treatment [29], etc. The main mechanism of adhesion is the mechanical and chemical interaction between the seed layer and the polymer. Yoong Oh et al. [30] studied the effect of plasma pre-treatment on the adhesion of Cu/Ti to the polymer dielectric. The formation of an interlayer between the titanium seed and ABF was reported. Transmission electron microscopy (TEM) images showed the formation of a few nanometers-thick inter-layer on a plasma-treated ABF dielectric [30]. There are numerous reports on the study of metal–polymer interactions. X-ray photoelectron spectroscopy (XPS) has proven to be an important tool for the study of these interactions [31,32,33,34,35,36,37]. Freilich et al. [35,36] studied the interactions of copper and titanium with polyimide using XPS and ultraviolet photoemission spectroscopy (UPS). Based on their findings, a mechanism for the formation of Ti–polymer bonds was proposed. It was theorized that the interaction of titanium with polyimide led to the formation of Ti-O bonds, followed by Ti–C bonds. The formation of reduced imide as an intermediate was also hypothesized. Burkstrand et al. [37] studied the interactions of evaporated Cu, Ni, and Cr on a variety of polymer substrates. The proposed mechanism consisted of the formation of chelate-like metal–oxygen–polymer complexes. The higher adhesion of metals with certain polymers was attributed to the presence of chelate-like complexes. The effects of different types of plasmas on polymer surfaces have been reported in the literature [30,32,34,38,39,40,41]. The breaking of polymer bonds due to plasma leads to the formation of active chemical species on the polymer surface. This improves the chemical interaction and wettability of the surface and, therefore, aids adhesion. Some reports have also indicated that the effect of plasma diminishes with duration and high temperature [41].

Some of the important processing steps that influence the interactions between metals and polymers are plasma surface treatment, deposition of metals, and annealing. Plasma treatment affects the polymer surface in two ways: it roughens the polymer surface and creates unsatisfied bonds, thereby activating the surface. During the deposition of metal, the arriving atoms may perform a random walk on the surface or diffuse into the polymer. Metal atoms encountering each other on their diffusion path may form aggregates at the surface and in the polymer bulk [42].

**Figure 8:**
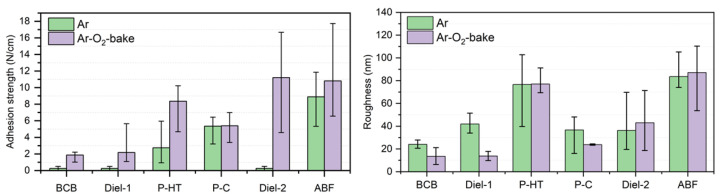
Adhesion and roughness values for different dielectrics [43].

**Figure 9:**
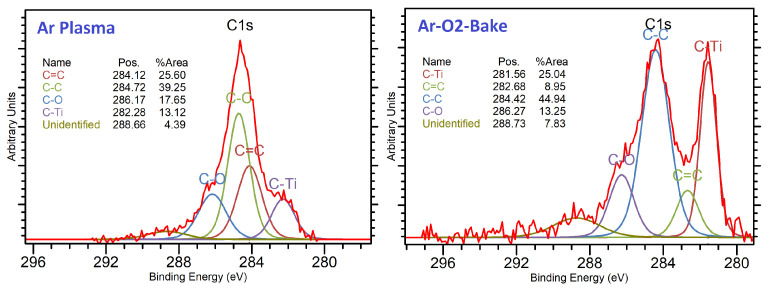
XPS C-1s spectra of ABF-GX92 showing the effect of plasma processes on the Ti–C interaction [43].

By optimizing the pre-sputtering processes, adhesion between the polymer and metal seed layers can be controlled as shown in Figure 8. A higher interaction between the metal and polymer is necessary. In the case of the titanium seed layer, the formation of a larger number of Ti–C bonds enhances adhesion. Figure 9 shows the difference in the XPS spectra of a titanium-deposited polymer dielectric. An increase in adhesion strength was observed from 8.9 N/cm to 11.2 N/cm for the ABF-GX92 dielectric. It corresponded to an 11.9% increase in the Ti–C component in the XPS C-1s spectra [43]. However, it should be noted that, because of the large variety of molecular groups present in polymers, different polymers may require different types of processing to optimize copper–polymer adhesion.

### 4.2. Thermal Cycling Reliability

**Figure 10:**
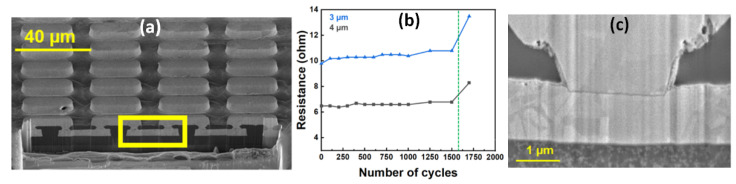
(**a**) Daisy chain structures formed using PID (**b**); resistance change with thermal cycling; (**c**) crack formation at microvia–pad interface [20].

Thermal cycling reliability is tested using the JEDEC (Joint Electron Device Engineering Council) standard [44]. For HPC applications, the RDL substrate is subject to temperature cycles from −55 °C to 125 °C. Due to the temperature variations, cyclic stresses are developed in the RDL, leading to fatigue failure in copper. Shinko electric industries have demonstrated an organic interposer with thin-film RDL [2]. The critical dimensions of the RDL in the integrated thin-film high-density organic package (i-THOP) were 2/2 μ m L/S and 10 μ m-diameter microvias. The thermal cycling reliability for 1000 thermal cycles and b-HAST reliability for 150 h were demonstrated. Kudo et al. demonstrated thermal cycling and HAST reliability of a nine-level polymer RDL structure with barrier layers [45,46]. Furuya et al. demonstrated a two-layer RDL structure with 2 μ m L/S and 5 μ m microvias in a polymer dielectric [47]. Hu et al. demonstrated a three-layer RDL structure with 1.5 μ m L/S and 10 μ m microvias using an embedded trench approach [48]. Nair et al. demonstrated the thermal cycling reliability of 4 μ m microvias using the embedded trench process [49]. Okamoto et al. demonstrated the thermal cycling reliability of 3 μ m microvias in a photosensitive polymer dielectric [20]. Figure 10a shows the daisy chain structures fabricated in a PID. Figure 10b shows the evolution of resistance over thermal cycles. The sharp increase in resistance at 1500 cycles was attributed to the cracking at the microvia–pad interface observed in Figure 10c.

**Figure 11:**
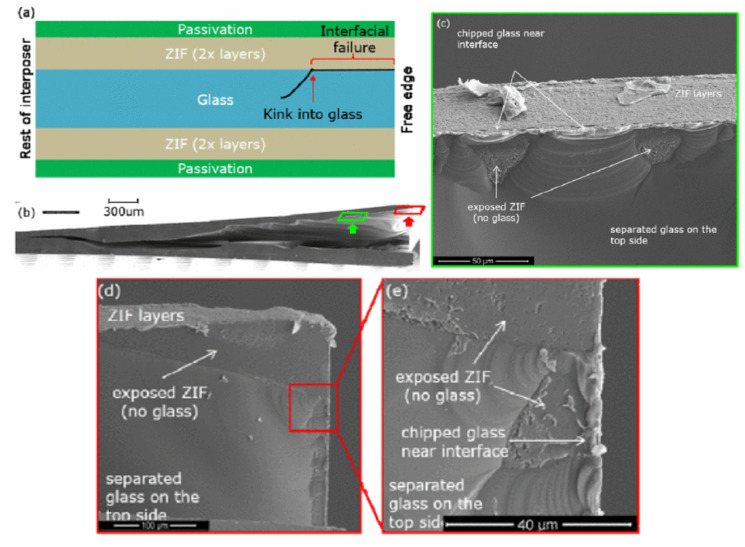
Cracking at polymer–glass interface after thermal cycling. (**a**) Schematic of stack up; (**b**) cracked glass specimen; (**c**) SEM image of cohesive cracking of glass; (**d**,**e**) SEM image of the corner of glass–polymer interface [50].

Glass-based interposer substrates are gaining importance due to their tunable CTE for optimizing board-level reliability [14]. However, because of the brittle nature of glass, cracking of the glass core is a challenge. Figure 11 shows cracking in a glass substrate at the polymer–glass interface. For the prevention of cracking in glass substrates, thinner, low-stress, low-CTE polymer dielectrics are needed to reduce the stresses on the glass. The optimization of the dicing parameters and pull-back mechanisms have also been shown to be helpful for the prevention of cracking [50].

### 4.3. Highly Accelerated Stress Test

**Figure 12:**
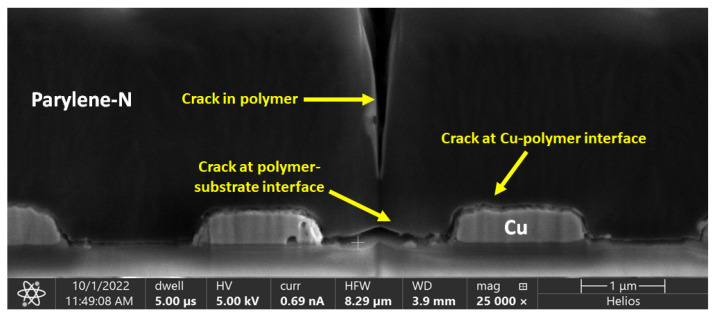
Cracking in Parylene-N due to moisture absorption during highly accelerated stress test.

The highly accelerated stress test (HAST), also known as the pressure cooker test, is critical for determining the reliability of RDL interposers. Moisture absorption by polymers can lead to mechanical and electrical failure in RDLs. Figure 12 shows cracking at various interfaces in Parylene-N due to high moisture uptake during HAST. The test samples were subject to an 85% relative humidity and a 135 °C temperature for 96 h. High moisture absorption is, thus, detrimental to the mechanical rigidity of polymer dielectrics. Low-moisture-absorbing polymers are, therefore, needed for preventing such failures.

### 4.4. Residual Stresses and Warpage

**Figure 13:**
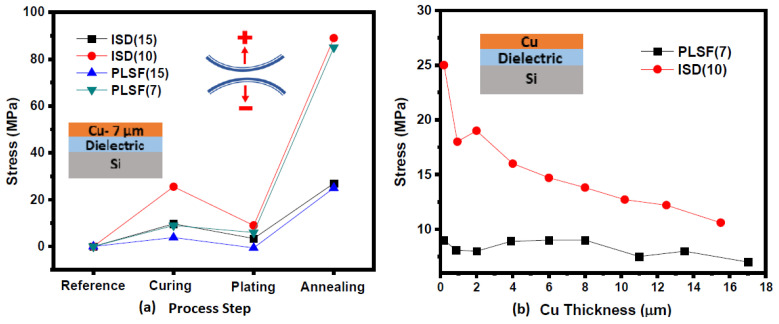
Residual stresses in RDLs (**a**) at various steps in fabrication and (**b**) for different copper thickness values [51].

Residual stress is developed in RDLs because of the thermal processes such as curing and annealing [51]. High residual stresses lead to large warpage of the substrate and can also induce cracks in the dielectric. Residual stress and warpage become critical issues when dealing with large-body interposers and substrates. Kovach et al. used low-stress processes such as electron-beam curing and electroplating to minimize the stress in the copper–polyimide layers [52]. Chen et al. studied the stress relaxation properties of polyimide in the metal–polyimide interface [53]. It was observed that an intermediate polyimide layer offers significant stress relaxation by plastic deformation. Electroplated copper has the most-pronounced effect on stress development due to its high elastic modulus. Electroplated copper undergoes self-annealing, leading to a gradual increase in stress over time [54,55]. Self-annealing of copper can also lead to the formation of voids due to stress migration [56,57]. Warpage of the substrate is proportional to the stress; thus, lowering stress would automatically lead to a lower warpage [58]. Warpage of interposers and substrates is important from the reliability and assembly point of view. Large warpage can lead to solder bridging during the assembly process and could also lead to poor reliability of solder joints due to accumulated stresses. Warpage depends on the modulus, CTE, and dimensions of the substrate. This was illustrated in a study by Hegde et al., wherein warpage was compared for different dielectric materials laminated on FR4. The material having the highest modulus showed the highest warpage, despite a low CTE value. The same study also showed that, when the properties of both the substrate and the dielectric were considered, the thickness, modulus, and CTE of the substrate influenced the warpage more strongly [59]. Figure 13 shows the stress evolution in RDLs with the process steps and copper thickness, respectively. Copper has the most-dominant effect on stress evolution, especially after annealing. Furthermore, with an increasing thickness of the copper, the stress becomes independent of the dielectric thickness and properties. However, the low modulus of the polymer dielectric helps keep the stress low for most parts of the fabrication process. The effects of the dielectric and substrate properties on stresses and warpage have been reported [60]. The elastic modulus and CTE of polymer dielectrics have the most-pronounced effect on the stresses and on RDL reliability.

## 5. Electrical Reliability

Electrical reliability concerns in polymer RDLs arise due to two main phenomena—ionic migration and dielectric breakdown. As we scale down the RDL dimensions, the electric field between two adjacent lines increases because of the reduction in the conductor spacing. The elevated electric field plays an important role in determining the electrical reliability of RDL L/S. Ionic impurities in the presence of moisture give rise to a higher ionic migration rate. Additionally, a higher electric field combined with elevated operating temperatures leads to leakage and dielectric breakdown. It is very crucial to address both of these challenges for achieving electrically reliable RDLs. Electrical reliability evaluations are conducted according to the JEDEC reliability standard [61].

### 5.1. Ionic Migration

**Figure 14:**
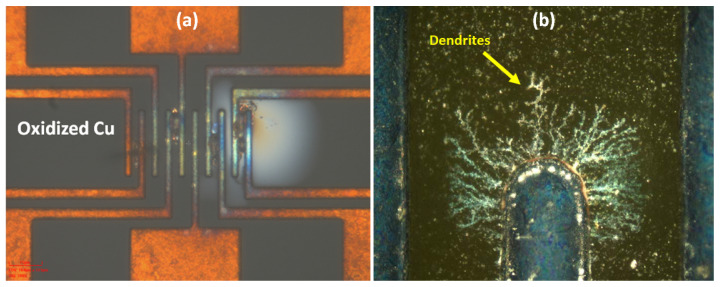
(**a**) Oxidation of Cu during the b-HAST of BCB-covered comb structures. (**b**) Dendrite formation due to ionic impurities [64].

Polymers contain ionic impurities originating from the byproducts during polymer synthesis [62,63]. Because of these impurities, polymer dielectrics act as electrolytes facilitating the transfer of metal ions across two conducting lines [64]. As we reduce the RDL L/S, the electric field across two lines increases and leads to an increase in the rate of migration of metal ions from the anode to the cathode. This can lead to the formation of conducting pathways or dendrites [65,66] across two conductors, leading to shorting. Figure 14a shows oxidation and shorting of comb structures coated with BCB dielectric. The test samples were subject to biased-HAST (b-HAST) conditions of an 85% relative humidity, 135 °C temperature, and 5 V applied bias for 96 h. Moisture absorption during b-HAST led to the oxidation of copper. The reaction of copper with oxygen from the polymer backbone is also a possibility. Figure 14b shows dendrite formation due to ionic impurities present in the dielectric. To prevent failures due to ionic migration, it is important to minimize ionic impurities arising from the polymerization reactions. Additionally, it is important to reduce moisture absorption by the careful selection of molecular groups during the formulation of polymer dielectrics.

### 5.2. Dielectric Breakdown and Leakage

**Figure 15:**
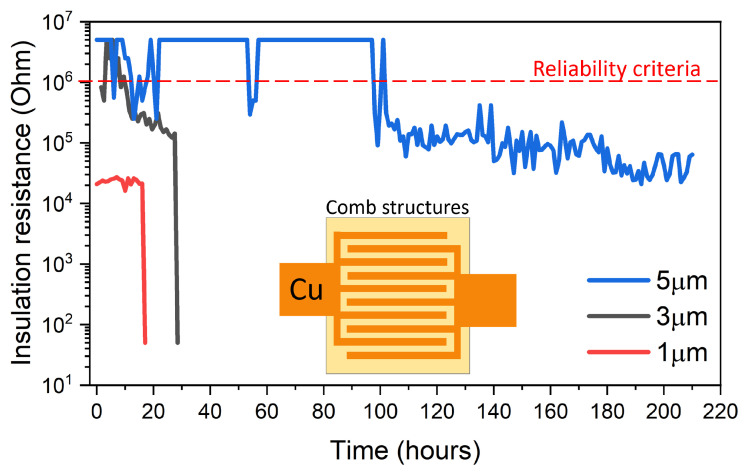
Effect of conductor spacing on insulation resistance of BCB-coated comb structures.

**Figure 16:**
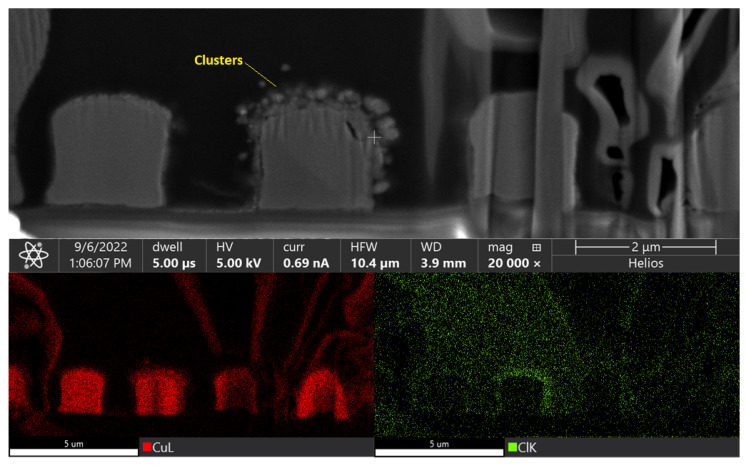
Dielectric breakdown in Parylene-C. EDS maps show the concentration of Cu and Cl around traces.

The dielectric breakdown strength is an important property for the electrical reliability of polymer RDLs. When a voltage is applied across a dielectric, the electrical insulation of the dielectric fails at a certain value of voltage, leading to a high leakage current. This phenomenon is known as “dielectric breakdown”. It is typically observed as an electrical arc across the electrodes, resulting in a catastrophic decrease in insulation resistance. The leakage current in polymer dielectrics does not follow Ohm’s law. Before the onset of breakdown, the current density across the electrodes increases almost exponentially with the electric field. Once reaching the breakdown potential, it abruptly increases to extremely high values, thus destroying the dielectric by burning due to localized high current densities. There are different mechanisms of dielectric breakdown reported in the literature—intrinsic, avalanche, thermal, hopping, charge-injection, and electro-mechanical breakdown [67,68,69,70]. Thermal breakdown occurs when the dielectric is overheated by an electric current, causing the polymer to melt or burn at a certain voltage. In this case, the dielectric strength is proportional to the square root of the plastic’s thermal and electrical conductivity ratio [68]. In this case, impact ionization is the most-common cause of electrical breakdown. The chemical and molecular structure of polymers affects the bond characteristics, as shown in Table 3, and thereby, the dielectric strength of polymers. The breakdown strength is directly proportional to the elastic modulus and inversely proportional to the dielectric constant [71]. Figure 15 shows the results of biased-HAST on BCB-coated comb structures. The test conditions were the same as mentioned earlier. The effect of conductor spacing on the failure time is clearly demonstrated. For a 5 μ m conductor spacing, failure did not occur even after 100 h, while for a 1 μ m spacing, failure occurred within 20 h. This was attributed to the dielectric breakdown of the polymer at elevated temperatures and at high electric fields. Figure 16 shows SEM images of electrical failure in comb structures with Parylene-C as the dielectric. The high leakage current caused localized melting of copper, leading to electrical shorting, possibly due to thermal breakdown. Energy dispersive spectroscopy (EDS) maps showed chlorine concentration and clustering around Cu traces, denoting accelerated failure due to the ionization of chlorine atoms.

Table 4 shows the dielectric constant and dielectric strength values of relevant dielectric materials used in packaging. Theoretically, a bias voltage of 530 V needs to be applied to cause dielectric breakdown across two conductors with a 1 μ m spacing and BCB as the dielectric. However, in the b-HAST experiments, the dielectric strength was found to be significantly lower than the values from the datasheet. This is because the breakdown strength is significantly degraded by the presence of defects and impurities. Furthermore, various other factors such as ramp rate, dielectric thickness, and temperature affect the measured dielectric strength values. Therefore, detailed studies need to be carried out to understand the effects of all these factors on dielectric breakdown, as well as on the electrical reliability of polymer dielectrics. With the miniaturization of critical dimensions in package RDLs, defect-free polymer dielectrics having high breakdown strengths are necessary for achieving electrical reliability.

## 6. Summary and Future Needs

The overall trend in the semiconductor industry is towards heterogeneous integration of chiplets onto interposers. Multiple high-bandwidth memory dies are expected to be integrated with logic dies all onto a single interposer. This requires larger interposer body sizes. This translates to performance, processing, and reliability challenges with respect to polymer dielectrics. The following list summarizes the critical needs.

- Lower D_*k*_ (<2.5) dielectrics are needed for achieving higher bandwidth densities, as well as for minimizing losses and latency. With larger interposer and substrate sizes, the total length of connections between chiplets is going to be longer than the traditional homogeneously integrated chips. This necessitates lower RDL capacitance for maintaining the electrical performance and loss budgets.
- Novel processing techniques are needed for integrating new dielectric materials. This needs to be performed using large panel-scale processing to lower the cost of larger substrates.
- Thinner dielectric layers (<5 μ m) are needed for reducing the overall buildup thickness. Larger substrates will have restrictively higher warpages with the current RDL design rules. It is critical to use thinner dielectrics and build thinner substrates. Additionally, thinner dielectrics are desired to make smaller and reliable microvias with diameters smaller than 5 μ m.
- Low-CTE (<40 ppm/K) and low-stress polymers are needed to minimize the stresses induced in RDLs. The miniaturization of RDLs will be restricted with the high stresses induced by current high-CTE dielectrics. The stability of CTE and mechanical properties up to the operating and solder reflow temperatures is also critical.
- Fillers in polymer dielectrics create processing, yield, and reliability challenges in achieving finer RDL dimensions, as discussed in Section 2. Filler-less polymer dielectrics with low roughness (Ra < 20 nm) are needed for the scaling of RDL lines, as well as microvias.
- Defect-free polymers with high dielectric breakdown strength are needed to prevent electrical failures in RDLs. Additionally, low moisture absorption (<0.1 wt%) and zero ionic content are critical to prevent ionic migration of metal atoms.

## Figures and Tables

**Table 1:** State-of-the-art in RDL technologies.

Technology	Package Architecture	Dielectric	Diel. Const.	Diel. Thickness ( μ m)	RDL L/S/via ( μ m)	Process
Shinko iTHOP [2,5]	2.5D organic interposer	Polymer	3.9	>5	2/2/10	Semi-additive
Amkor SWIFT [6,7]	Fan-out (wafer)	Polyimide	3–3.6	>15	2/2/10	Semi-additive
SEMCO [8]	Fan-out (panel)	PBO	3.1	>5	2/2/6	Semi-additive
Kyocera APX [3,9]	2.5D organic interposer	Epoxy	3.1	>8	6/6/15	Semi-additive
DNP [10]	2.5D glass interposer	Polyimide	3–3.6	>12	2/2/20	Semi-additive
Amkor SLIM [11]	2.5D interposer	SiO_2_	4	>2	2/2/2	Damascene
Intel EMIB [1]	Si bridge	SiO_2_	4	>2	2/2/2	Damascene
TSMC CoWoS [4]	2.5D Si interposer	SiO_2_	4	>2	0.5/0.5	Damascene

**Table 2:** Survey of commercially available polymer dielectrics.

	Properties	Manufacturer	Dk	Df	Film Type	Elastic Modulus (GPa)	CTE (ppm/K)	Max. Elongation (%)	Tensile Strength (MPa)	Curing Temp. (°C)	Glass Trans. Temp. (°C)	Moisture Abs. (%)
Dielectric												
ABF GX92		Ajinomoto	3.2	0.017	DF (>5 μ m)	5	39	5.6	98	180	168	<1
ABF GX-T31		Ajinomoto	3.4	0.014	DF (>15 μ m)	7.5	23	2.4	104	170	172	<0.6
ABF GX-E4		Ajinomoto	3.4	0.0093	DF (>15 μ m)	13	12	0.8	98	-	180	<0.4
ABF GX-E5		Ajinomoto	3.3	0.0073	DF (>15 μ m)	17	10	0.8	106	-	212	<0.4
ABF GX13		Ajinomoto	3.1	0.019	DF (>15 μ m)	4	46	5	93	-	177	<1.1
ABF GZ22		Ajinomoto	3.2	0.011	DF (>15 μ m)	6.4	31	3.2	116	-	192	<0.6
ABF GZ41		Ajinomoto	3.3	0.0074	DF (>15 μ m)	9	20	1.7	120	-	198	<0.5
ABF GY11		Ajinomoto	3.2	0.0042	DF (>15 μ m)	8.9	26	3.2	115	-	165	<0.2
ALX		AGC	2.6	0.003	liquid	2.4	60	30	100	190	230	<0.4
AM-270		Asahi Kasei	2.9	-	liquid	2.7	50–60	60	>120	350	300	0.6
MA-1000		Asahi Kasei	3.9	-	liquid	3.6	30–40	50	>120	220	240	1.8
BL-300		Asahi Kasei	3.3	-	liquid	3.4–3.5	40–70	50	>120	200–350	200–260	0.8
BM-300		Asahi Kasei	3.3	-	liquid	4.8–5.8	20–60	10–30	>150	200–350	220–390	0.8
I-8100		Asahi Kasei	3.3	-	liquid	3.3	40–50	50	>150	350	290	0.8
Cyclotene 4000		Dow	2.65	0.008	liquid	2.9	42	13	96	250	-	<0.2
Cyclotene P6001		Dow	3	0.009	liquid	3.6	55	-	-	390	-	1
Cyclotene 6505		Dow	3.2	0.015	liquid	2.9	45	-	-	390	-	1.1
Durimide 7300		Fujifilm	3.2–3.3	-	liquid	2.5	55	85	215	-	285	1.08
Durimide 100		Fujifilm	3.1–3.4	0.006	liquid	3.3	32	80	260	-	371	1.7
HD-4100		HD Microsys.	3.36	0.001	liquid	3.4	35	45	200	375	330	-
HD-8820		HD Microsys.	2.94	0.0089	liquid	2.6	52	66	149	350	306	<0.5
HD 8930 PBO		HD Microsys.	3.1	0.010	liquid	1.8	80	80	170	250	240	1.5
HD 8940 PBO		HD Microsys.	2.9	0.009	liquid	2.2	60	100	170	250	230	1.5
PI-2574		HD Microsys.	3.3	0.002	liquid	2.45	40	10	131	300	320	2-3
PI-2545		HD Microsys.	3.3	0.002	liquid	2.3	13	100	260	350	400	3.1
PI-2611		HD Microsys.	2.9	0.002	liquid	8.5	3	100	350	350	360	0.5
WPR-5200		JSR	3.5	-	liquid	2.5	54	6.5	80	200	-	-
HC-F		JSR	2.49	0.0016	liquid	1.1	66	60	48	185	138	0.3
PRL-29		Kayaku Adv. Mat.	2.5	0.004	liquid	1.8	62	35	60	200	220	0.03
KMSF-1000		Kayaku Adv. Mat.	2.6	0.008	liquid	0.14	140	160	37	175	57	0.1
KMSF-2000		Kayaku Adv. Mat.	2.5	0.003	liquid	1.6	60	65	60	200	215	0.03
Vecstar CTQ-50		Kuraray	3.3	0.002	DF (>25 μ m)	3.6	15	30	180	-	-	0.04
Vecstar CTF-50		Kuraray	3.3	0.002	DF (>25 μ m)	3.1	18	40	190	-	0.04	
NC0201		Namics	2.5	0.0025	DF (>5 μ m)	0.8	130	-	-	200	185	-
Parylene-N		SCS	2.65	0.0006	vapor	2.4	69	<250	48	-	160	<0.1
Parylene-C		SCS	2.95	0.013	vapor	2.8	35	<200	69	-	125	<0.1
Parylene-D		SCS	2.8	0.0002	vapor	2.6	38	<200	76	-	125	<0.1
Parylene-HT		SCS	2.17	0.001	vapor	2.6	36	<200	52	-	377	<0.01
Chemfilm TH-012		Saint Gobain	3.3	0.005	DF (13 μ m)	3.1	40	85	245	-	>380	2.5
NQ07X		Sekisui Chem.	3.3	0.0037	DF (>20 μ m)	10	27	2.6	105	-	183	-
NX04H		Sekisui Chem.	3.3	0.009	DF (>20 μ m)	8.0	24.5	2.4	100	-	205	-
NR11		Sekisui Chem.	3.4	0.008	DF (15 μ m)	12.5	17	-	-	-	195	-
NR50		Sekisui Chem.	3.3	0.015	DF (10 μ m)	5.4	44	-	-	-	194	-
BL α -3700GS		Sumitomo Bakelite	3.1	0.012	-	5	35	-	-	-	-	1

**Table 3:** Characteristics of common chemical bonds present in polymers [25].

Bond	Polarizability (A^3^)	Bond Strength (kcal/mol)
C-C	0.531	83
C-F	0.555	116
C-O	0.584	84
C-H	0.652	99
O-H	0.706	102
C=O	1.02	176
C=C	1.643	146

**Table 4:** Breakdown strength values of various dielectrics.

Dielectric	Dielectric Const.	Breakdown Strength (MV/m)	Ref.
Benzocyclobutene (BCB)	2.65	530	[72]
Parylene-C	2.95	220	[73]
Parylene-HT	2.17	213	[73]
Parylene-N	2.65	275	[73]
Parylene-D	2.8	217	[73]
Polybenzoxazoles (PBO)	2.9–3.3	150–470	[74]
Polyimide (PI)	2.9	470	[75]
Liquid crystal polymer (LCP)	3.3	200	[76]
Polytetrafluoroethylene (PTFE)	2–2.1	370–742	[77]
SiO_2_	3.9	430–810	[78]

## References

[1] Mahajan R., Qian Z., Viswanath R.S., Srinivasan S., Aygün K., Jen W.L., Sharan S., Dhall A. (2019). Embedded multidie interconnect bridge—A localized, high-density multichip packaging interconnect. IEEE Trans. Compon. Packag. Manuf. Technol..

[2] Oi K., Otake S., Shimizu N., Watanabe S., Kunimoto Y., Kurihara T., Koyama T., Tanaka M., Aryasomayajula L., Kutlu Z. (2014). Development of new 2.5 D package with novel integrated organic interposer substrate with ultra-fine wiring and high density bumps. Proceedings of the 2014 IEEE 64th Electronic Components and Technology Conference (ECTC).

[3] Yamada T. (2015). Organic Interposer and Embedded Substrate. Proceedings of the 2015 Packaging Symposium.

[4] Hou S., Chen W.C., Hu C., Chiu C., Ting K., Lin T., Wei W., Chiou W., Lin V.J., Chang V.C. (2017). Wafer-level integration of an advanced logic-memory system through the second-generation CoWoS technology. IEEE Trans. Electron Devices.

[5] Tsukamoto K., Kajiki A., Kunimoto Y., Mizuno M., Nakamura M., Nakazawa S., Koyama T. (2019). Analysis on signal and power integrity of 2.3 D structure organic package. Int. Symp. Microelectron..

[6] Lee K. High-density fan-out technology for advanced SiP and 3D heterogeneous integration. Proceedings of the 2018 IEEE International Reliability Physics Symposium (IRPS).

[7] Zwenger C., Huemoeller R., Kim J., Kim D., Do W., Seo S. (2015). Silicon wafer integrated fan-out technology. Addit. Pap. Present..

[8] Kim J., Choi I., Park J., Lee J.E., Jeong T., Byun J., Ko Y., Hur K., Kim D.W., Oh K.S. (2018). Fan-out panel level package with fine pitch pattern. Proceedings of the 2018 IEEE 68th Electronic Components and Technology Conference (ECTC).

[9] Ishida M. APX (Advanced Package X)-Advanced organic technology for 2.5 D interposer. Proceedings of the 2014 CPMT Seminar, Latest Advances in Organic Interposers.

[10] Kudo H., Aritsuka Y., Masaya T., Kasai R., Suyama J., Takeda M., Okazaki Y., Iida H., Kitayama D., Sakamoto K. (2018). Introduction of Sub-2-micron Cu traces to EnCoRe enhanced copper redistribution layers for heterogeneous chip integration. Proceedings of the 2018 International Conference on Electronics Packaging and iMAPS All Asia Conference (ICEP-IAAC).

[11] Huemoeller R. (2015). Amkor’s SLIM & SWIFT Package Technology. Amkor Technology, SVP Advanced Package Technology Develop & IP. https://www.3dincites.com/wp-content/uploads/slim-swift-customer-overview-may-13-2015.pdf.

[12] Liu F., Nimbalkar P., Aslani-Amoli N., Kathaperumal M., Tummala R., Swaminathan M. (2023). A Critical Review of Lithography Methodologies and Impacts of Topography on 2.5 D/3D Interposers. IEEE Trans. Compon. Packag. Manuf. Technol..

[13] Liu F., Zhang R., DeProspo B.H., Dwarakanath S., Nimbalkar P., Ravichandran S., Weyers D., Kathaperumal M., Tummala R.R., Swaminathan M. Advances in High Performance RDL Technologies for Enabling IO Density of 500 IOs/mm/layer and 8-micron IO Pitch Using Low-k Dielectrics. Proceedings of the 2020 IEEE 70th Electronic Components and Technology Conference (ECTC).

[14] Tummala R., Deprospo B., Dwarakanath S., Ravichandran S., Nimbalkar P., Nedumthakady N., Swaminathan M. Glass Panel Packaging, as the Most Leading-Edge Packaging: Technologies and Applications. Proceedings of the 2020 Pan Pacific Microelectronics Symposium (Pan Pacific).

[15] Nimbalkar P., Aguebor M., Kathaperumal M., Swaminathan M., Tummala R. Evaluation of Parylene-HT as Dielectric for Application in Advanced Package Substrates. Proceedings of the 2023 IEEE 73rd Electronic Components and Technology Conference (ECTC).

[16] Tummala R., Swaminathan M., Nimbalkar P. (2022). A new and historic packaging era. Chip Scale Rev..

[17] DeProspo B. (2020). Modeling, Design and Demonstration of 1 μm Wide Low Resistance Panel Redistribution Layer Technology for High Performance Computing Applications. Ph.D. Thesis.

[18] Nimbalkar P., Kathaperumal M., Liu F., Swaminathan M., Tummala R. Reliability Modeling of Micro-vias in High-Density Redistribution Layers. Proceedings of the 2021 IEEE 71st Electronic Components and Technology Conference (ECTC).

[19] Liu F., Ito H., Zhang R., DeProspo B.H., Benthaus F., Akimaru H., Hasegawa K., Sundaram V., Tummala R.R. (2018). Low cost panel-based 1-2 micron RDL technologies with lower resistance than Si BEOL for large packages. Proceedings of the 2018 IEEE 68th Electronic Components and Technology Conference (ECTC).

[20] Okamoto D., Shibasaki Y., Shibata D., Hanada T., Liu F., Kathaperumal M., Tummala R.R. (2019). Fabrication and Reliability Demonstration of 3 μm Diameter Photo Vias at 15 μm Pitch in Thin Photosensitive Dielectric Dry Film for 2.5 D Glass Interposer Applications. Proceedings of the 2019 IEEE 69th Electronic Components and Technology Conference (ECTC).

[21] Nimbalkar P., Bhaskar P., Blancher C., Kathaperumal M., Swaminathan M., Tummala R. (2022). Novel zero side-etch process for < 1 μm package redistribution layers. Proceedings of the 2022 IEEE 72nd Electronic Components and Technology Conference (ECTC).

[22] Nair C. (2019). Modeling, Design, Materials, Processes and Reliability of Multi-Layer Redistribution Wiring Layers on Glass Substrates for Next Generation of High-Performance Computing Applications. Ph.D. Thesis.

[23] Swaminathan M., Ravichandran S. (2022). Heterogeneous Integration for AI application: Status and future needs. Chip Scale Rev..

[24] Wei J., Zhu L. (2020). Intrinsic polymer dielectrics for high energy density and low loss electric energy storage. Prog. Polym. Sci..

[25] Kohl P.A. (2011). Low–dielectric constant insulators for future integrated circuits and packages. Annu. Rev. Chem. Biomol. Eng..

[26] Polymers in Electronic Packaging: Build-Up Films for Flip Chip Semiconductor Substrates, Part Two. https://polymerinnovationblog.com/polymers-in-electronic-packaging-build-up-films-for-flip-chip-semiconductor-substrates-part-two/.

[27] Sundaram V. (2009). Advances in Electronic Packaging Technologies by Ultra-Small Microvias, Super-Fine Interconnections and Low Loss Polymer Dielectrics. Ph.D. Thesis.

[28] Horiuchi M., Yamasaki T., Shimizu Y. (2010). Metallization Technologies on a Smooth Resin Surface for the Next Generation of Flip Chip Packaging. Trans. Jpn. Inst. Electron. Packag..

[29] Inoue K., Matsui K., Watanabe M., Honma H. (2008). Surface Modification of Polyimide Using UV Light and Formation of Circuit Patterns. J. Surf. Finish. Soc. Jpn..

[30] Oh Y., Kim E.J., Kim Y., Choi K., Han W.B., Kim H.S., Yoon C.S. (2016). Adhesion of sputter-deposited Cu/Ti film on plasma-treated polymer substrate. Thin Solid Film..

[31] Friedrich J.F., Koprinarov I., Giebler R., Lippitz A., Unger W.E.S. (1999). Reactions and Intermediates at the Metal-Polymer Interface as Observed by XPS and NEXAFS Spectroscopy. J. Adhes..

[32] Kim S.H., Na S.W., Lee N.E., Nam Y.W., Kim Y.H. (2005). Effect of surface roughness on the adhesion properties of Cu/Cr films on polyimide substrate treated by inductively coupled oxygen plasma. Surf. Coat. Technol..

[33] Kim S.H., Cho S.H., Lee N.E., Kim H.M., Nam Y.W., Kim Y.H. (2005). Adhesion properties of Cu/Cr films on polyimide substrate treated by dielectric barrier discharge plasma. Surf. Coat. Technol..

[34] Paik K.W., Cole H.S., Saia R.J., Chera J.J. (1993). Studies on metal/benzocyclobutene (BCB) interface and adhesion. J. Adhes. Sci. Technol..

[35] Freilich S., Ohuchi F. (1987). Reactions at the polyimide-metal interface. Polymer.

[36] Ohuchi F.S., Freilich S.C. (1986). Metal polyimide interface: A titanium reaction mechanism. J. Vac. Sci. Technol. A.

[37] Burkstrand J.M. (1981). Metal-polymer interfaces: Adhesion and x-ray photoemission studies. J. Appl. Phys..

[38] Nakamura Y., Suzuki Y., Watanabe Y. (1996). Effect of oxygen plasma etching on adhesion between polyimide films and metal. Thin Solid Film..

[39] Lee S.B., Kim Y.K. (2009). Adhesion Improvement of Polyimide/Metal Interface by He/O_2_/NF_3_ Atmospheric Pressure Plasma. Plasma Process. Polym..

[40] Nguyen T.P., Lahmar A., Jonnard P. (1998). Adhesion Improvement of Poly(Phenylene-Vinylene) Substrates Induced by Argon-Oxygen Plasma Treatment. J. Adhes..

[41] Egitto F.D., Matienzo L.J. (1994). Plasma modification of polymer surfaces for adhesion improvement. IBM J. Res. Dev..

[42] Faupel F., Zaporojtchenko V., Thran A., Strunskus T., Kiene M., Gupta D. (2005). 7-Metal Diffusion in Polymers and on Polymer Surfaces. Diffusion Processes in Advanced Technological Materials.

[43] Nimbalkar P., Blancher C., Kathaperumal M., Swaminathan M., Tummala R. (2022). Effect of titanium-polymer interactions on adhesion of polymer-copper redistribution layers in advanced packaging. IEEE Trans. Device Mater. Reliab..

[44] Jedec Solid State Technology Association (2009). JEDEC Standard-Temperature Cycling-JESD22-A104D.

[45] Kudo H., Takano T., Tanaka M., Kasai R., Suyama J., Akazawa M., Takeda M., Mawatari H., Sasao T., Okazaki Y. (2016). A Characterized Redistribution Layer Architecture for Advanced Packaging Technologies. Proceedings of the 2016 IEEE 66th Electronic Components and Technology Conference (ECTC).

[46] Kudo H., Kasai R., Suyama J., Takeda M., Okazaki Y., Iida H., Kitayama D., Sasao T., Sakamoto K., Sato H. (2017). Demonstration of high electrical reliability of sub-2 Micron Cu traces covered with inorganic dielectrics for advanced packaging technologies. Proceedings of the 2017 IEEE 67th Electronic Components and Technology Conference (ECTC).

[47] Furuya R., Lu H., Liu F., Deng H., Ando T., Sundaram V., Tummala R. (2015). Demonstration of 2 μm RDL wiring using dry film photoresists and 5 μm RDL via by projection lithography for low-cost 2.5 D panel-based glass and organic interposers. Proceedings of the 2015 IEEE 65th Electronic Components and Technology Conference (ECTC).

[48] Hu D.C., Yeh W.L., Chen Y.H., Tain R. (2016). 2/2um Embedded Fine Line Technology for Organics Interposer Applications. Proceedings of the 2016 IEEE 66th Electronic Components and Technology Conference (ECTC).

[49] Nair C., DeProspo B., Hichri H., Arendt M., Liu F., Sundaram V., Tummala R. (2018). Reliability Studies of Excimer Laser-Ablated Microvias Below 5 Micron Diameter in Dry Film Polymer Dielectrics for Next Generation, Panel-Scale 2.5 D Interposer RDL. Proceedings of the 2018 IEEE 68th Electronic Components and Technology Conference (ECTC).

[50] McCann S., Sato Y., Sundaram V., Tummala R.R., Sitaraman S.K. (2015). Prevention of cracking from RDL stress and dicing defects in glass substrates. IEEE Trans. Device Mater. Reliab..

[51] Nimbalkar P., Liu F., Watanabe A., Weyers D., Kathaperumal M., Lin C.P., Naohito F., Makita T., Watanabe N., Kubo A. Fabrication and reliability demonstration of 5 μm redistribution layer using low-stress dielectric dry film. Proceedings of the 2020 IEEE 70th Electronic Components and Technology Conference (ECTC).

[52] Kovach D., Amirgulyan N., CHIEN C.P., Tanielian M. (2000). Minimizing stress in Cu/polyimide processes for large format MCM manufacturing. Int. J. Microcircuits Electron. Packag..

[53] Chen S., Yang C., Faupel F., Ho P. (1988). Stress relaxation during thermal cycling in metal/polyimide layered films. J. Appl. Phys..

[54] Lagrange S., Brongersma S., Judelewicz M., Saerens A., Vervoort I., Richard E., Palmans R., Maex K. (2000). Self-annealing characterization of electroplated copper films. Microelectron. Eng..

[55] Hara T., Toida H., Shimura Y. (2003). The self-annealing phenomenon in copper interconnection. Electrochem. Solid State Lett..

[56] Matsuyama H., Suzuki T., Nakamura T., Shiozu M., Ehara H., Oshima M., Soeda T., Hosoi H., Yamabe K. (2017). Voiding generation in copper interconnect under room temperature storage in 12 years. Jpn. J. Appl. Phys..

[57] Matsuyama H., Suzuki T., Shiozu M., Ehara H., Soeda T., Hosoi H., Oshima M., Yamabe K. (2019). Verification of Copper Stress Migration Under Low Temperature Long Time Stress. Proceedings of the 2019 IEEE International Reliability Physics Symposium (IRPS).

[58] Zhu C., Ning W., Xu G., Luo L. (2014). Stress evolution during thermal cycling of copper/polyimide layered structures. Mater. Sci. Semicond. Process..

[59] Hegde S., Pucha R.V., Sitaraman S.K. (2004). Enhanced reliability of high-density wiring (HDW) substrates through new base substrate and dielectric materials. J. Mater. Sci. Mater. Electron..

[60] Mahalingam S., Hegde S., Ramakrishna G., Pucha R.V., Sitaraman S.K. Material interaction effects in the reliability of high density interconnect (HDI) boards. Proceedings of the ASME International Mechanical Engineering Congress and Exposition.

[61] (2009). Highly Accelerated Temperature and Humidity Stress Test (HAST).

[62] Oka Y., Koizumi N. (1982). Effects of impurity ions on electrical properties of poly (vinylidene fluoride). Polym. J..

[63] Egginger M., Schwoediauer R. (2012). Analysis of mobile ionic impurities in polyvinylalcohol thin films by thermal discharge current and dielectric impedance spectroscopy. AIP Adv..

[64] Bumiller E., Hillman C. (2009). A review of models for time-to-failure due to metallic migration mechanisms. White Paper Issued by DfR Solutions.

[65] Lambert D., Gannamani R., Blish R. (2004). Dendrite fuse re-growth kinetics on organic substrates for microprocessors. Proceedings of the 2004 IEEE International Reliability Physics Symposium.

[66] Kim S.A., Ahn D.S., Eum Y.H., Kim D.H., Kim Y.B. (2008). Case study of copper dendrite growth under HAST test. Proceedings of the 2008 15th International Symposium on the Physical and Failure Analysis of Integrated Circuits.

[67] Zhao L., Liu C.L. (2020). Review and mechanism of the thickness effect of solid dielectrics. Nanomaterials.

[68] Karlsson M. (2014). Investigation of the Dielectric Breakdown Strength of Polymer Nanocomposites. https://www.diva-portal.org/smash/record.jsf?pid=diva2%3A731544&dswid=9814.

[69] Ho J., Jow T.R. (2012). High field conduction in biaxially oriented polypropylene at elevated temperature. IEEE Trans. Dielectr. Electr. Insul..

[70] Ju T., Chen X., Langhe D., Ponting M., Baer E., Zhu L. (2022). Enhancing breakdown strength and lifetime of multilayer dielectric films by using high temperature polycarbonate skin layers. Energy Storage Mater..

[71] Qiao R., Wang C., Chen S., He G., Liu Z., Luo H., Zhang D. (2022). High-temperature dielectric polymers with high breakdown strength and energy density via constructing the electron traps in blends. Compos. Part Appl. Sci. Manuf..

[72] Dow Cyclotene 4000 Series Advanced Electronic Resins. https://kayakuam.com/wp-content/uploads/2019/10/cyclotene_4000_immersion_dev.pdf.

[73] Specialty Coating Systems (SCS) (2016). SCS Parylene Properties.

[74] Gotro J. Polymers in Electronics Part Seven: Redistribution Layers for Fan-Out Wafer Level Packaging. https://polymerinnovationblog.com/polymers-electronics-part-seven-redistribution-layers-fan-wafer-level-packaging/.

[75] Microsystems H. HD-8820 Aqueous Positive Polyimide. https://rndmate.com/download?path=request_attachments/HD-8820_ProcessGuide.pdf.

[76] Kuraray. https://www.kuraray.eu/fileadmin/industry_solutions/electronics/downloads/kuraray_FCCL_Brochure_EN_0531ol.pdf.

[77] Kerwien C.M., Malandro D.L., Broomall J.R. (2016). Large area DC dielectric breakdown voltage measurement of BOPP and PTFE thin films. Proceedings of the 2016 IEEE Conference on Electrical Insulation and Dielectric Phenomena (CEIDP).

[78] Bartzsch H., Glöß D., Frach P., Gittner M., Schultheiß E., Brode W., Hartung J. (2009). Electrical insulation properties of sputter-deposited SiO_2_, Si_3_N_4_ and Al_2_O_3_ films at room temperature and 400 C. Phys. Status Solidi (A).

