# Correction: Nimbalkar et al. A Review of Polymer Dielectrics for Redistribution Layers in Interposers and Package Substrates. *Polymers* 2023, *15*, 3895

**DOI:** 10.3390/polym16192751

**Published:** 2024-09-29

**Authors:** Pratik Nimbalkar, Pragna Bhaskar, Mohanalingam Kathaperumal, Madhavan Swaminathan, Rao R. Tummala

**Affiliations:** 1 3D Systems Packaging Research Center, Georgia Institute of Technology, Atlanta, GA 30332, USA; pragna@gatech.edu (P.B.); kmohan@ece.gatech.edu (M.K.); rtummala@ece.gatech.edu (R.R.T.); 2 Department of Electrical Engineering, Pennsylvania State University, University Park, PA 16802, USA; mvs7249@psu.edu

## Error in Table

In the original publication [1], there was a mistake in Table 2 as published. The values of Tensile Strength with respect to Parylene-N, Parylene-C, Parylene-D, and Parylene-HT were incorrect. The corrected Table 2 appears below. The authors state that the scientific conclusions are unaffected. This correction was approved by the Academic Editor. The original publication has also been updated [1].

## Figures and Tables

**Table 2:** Survey of commercially available polymer dielectrics.

	Properties	Manufacturer	Dk	Df	Film Type	Elastic Modulus (GPa)	CTE (ppm/K)	Max. Elongation (%)	Tensile Strength (MPa)	Curing Temp. (°C)	Glass Trans. Temp. (°C)	Moisture Abs. (%)
Dielectric												
ABF GX92		Ajinomoto	3.2	0.017	DF (>5 μm)	5	39	5.6	98	180	168	<1
ABF GX-T31		Ajinomoto	3.4	0.014	DF (>15 μm)	7.5	23	2.4	104	170	172	<0.6
ABF GX-E4		Ajinomoto	3.4	0.0093	DF (>15 μm)	13	12	0.8	98	-	180	<0.4
ABF GX-E5		Ajinomoto	3.3	0.0073	DF (>15 μm)	17	10	0.8	106	-	212	<0.4
ABF GX13		Ajinomoto	3.1	0.019	DF (>15 μm)	4	46	5	93	-	177	<1.1
ABF GZ22		Ajinomoto	3.2	0.011	DF (>15 μm)	6.4	31	3.2	116	-	192	<0.6
ABF GZ41		Ajinomoto	3.3	0.0074	DF (>15 μm)	9	20	1.7	120	-	198	<0.5
ABF GY11		Ajinomoto	3.2	0.0042	DF (>15 μm)	8.9	26	3.2	115	-	165	<0.2
ALX		AGC	2.6	0.003	liquid	2.4	60	30	100	190	230	<0.4
AM-270		Asahi Kasei	2.9	-	liquid	2.7	50–60	60	>120	350	300	0.6
MA-1000		Asahi Kasei	3.9	-	liquid	3.6	30–40	50	>120	220	240	1.8
BL-300		Asahi Kasei	3.3	-	liquid	3.4–3.5	40–70	50	>120	200–350	200–260	0.8
BM-300		Asahi Kasei	3.3	-	liquid	4.8–5.8	20–60	10–30	>150	200–350	220–390	0.8
I-8100		Asahi Kasei	3.3	-	liquid	3.3	40–50	50	>150	350	290	0.8
Cyclotene 4000		Dow	2.65	0.008	liquid	2.9	42	13	96	250	-	<0.2
Cyclotene P6001		Dow	3	0.009	liquid	3.6	55	-	-	390	-	1
Cyclotene 6505		Dow	3.2	0.015	liquid	2.9	45	-	-	390	-	1.1
Durimide 7300		Fujifilm	3.2–3.3	-	liquid	2.5	55	85	215	-	285	1.08
Durimide 100		Fujifilm	3.1–3.4	0.006	liquid	3.3	32	80	260	-	371	1.7
HD-4100		HD Microsys.	3.36	0.001	liquid	3.4	35	45	200	375	330	-
HD-8820		HD Microsys.	2.94	0.0089	liquid	2.6	52	66	149	350	306	<0.5
HD 8930 PBO		HD Microsys.	3.1	0.010	liquid	1.8	80	80	170	250	240	1.5
HD 8940 PBO		HD Microsys.	2.9	0.009	liquid	2.2	60	100	170	250	230	1.5
PI-2574		HD Microsys.	3.3	0.002	liquid	2.45	40	10	131	300	320	2-3
PI-2545		HD Microsys.	3.3	0.002	liquid	2.3	13	100	260	350	400	3.1
PI-2611		HD Microsys.	2.9	0.002	liquid	8.5	3	100	350	350	360	0.5
WPR-5200		JSR	3.5	-	liquid	2.5	54	6.5	80	200	-	-
HC-F		JSR	2.49	0.0016	liquid	1.1	66	60	48	185	138	0.3
PRL-29		Kayaku Adv. Mat.	2.5	0.004	liquid	1.8	62	35	60	200	220	0.03
KMSF-1000		Kayaku Adv. Mat.	2.6	0.008	liquid	0.14	140	160	37	175	57	0.1
KMSF-2000		Kayaku Adv. Mat.	2.5	0.003	liquid	1.6	60	65	60	200	215	0.03
Vecstar CTQ-50		Kuraray	3.3	0.002	DF (>25 μm)	3.6	15	30	180	-	-	0.04
Vecstar CTF-50		Kuraray	3.3	0.002	DF (>25 μm)	3.1	18	40	190	-	0.04	
NC0201		Namics	2.5	0.0025	DF (>5 μm)	0.8	130	-	-	200	185	-
Parylene-N		SCS	2.65	0.0006	vapor	2.4	69	<250	48	-	160	<0.1
Parylene-C		SCS	2.95	0.013	vapor	2.8	35	<200	69	-	125	<0.1
Parylene-D		SCS	2.8	0.0002	vapor	2.6	38	<200	76	-	125	<0.1
Parylene-HT		SCS	2.17	0.001	vapor	2.6	36	<200	52	-	377	<0.01
Chemfilm TH-012		Saint Gobain	3.3	0.005	DF (13 μm)	3.1	40	85	245	-	>380	2.5
NQ07X		Sekisui Chem.	3.3	0.0037	DF (>20 μm)	10	27	2.6	105	-	183	-
NX04H		Sekisui Chem.	3.3	0.009	DF (>20 μm)	8.0	24.5	2.4	100	-	205	-
NR11		Sekisui Chem.	3.4	0.008	DF (15 μm)	12.5	17	-	-	-	195	-
NR50		Sekisui Chem.	3.3	0.015	DF (10 μm)	5.4	44	-	-	-	194	-
BLα-3700GS		Sumitomo Bakelite	3.1	0.012	-	5	35	-	-	-	-	1
